# Supplementary material for: Heparin-based hydrogel scaffolding alters the transcriptomic profile and increases the chemoresistance of MDA-MB-231 triple-negative breast cancer cells
Source: Biomater Sci. 2020 Feb 13;8(10):2786–96. doi: 10.1039/c9bm01481k (PMC7497406; doi:10.1039/c9bm01481k)
Supplement: Supplementary file 2 [file BM-008-C9BM01481K-s002.zip › Supplementary File 4/EGFvControl/Pathways/my_analysis.Gsea.1545200981068/HALLMARK_INTERFERON_GAMMA_RESPONSE.html]

Details for gene set HALLMARK\_INTERFERON\_GAMMA\_RESPONSE[GSEA]

|  || Dataset | expr.class.cls#EGF\_versus\_CONTROL.class.cls#EGF\_versus\_CONTROL\_repos |
| Phenotype | class.cls#EGF\_versus\_CONTROL\_repos |
| Upregulated in class | CONTROL |
| GeneSet | HALLMARK\_INTERFERON\_GAMMA\_RESPONSE |
| Enrichment Score (ES) | -0.43881312 |
| Normalized Enrichment Score (NES) | -2.1224258 |
| Nominal p-value | 0.0 |
| FDR q-value | 2.857143E-4 |
| FWER p-Value | 0.001 |
Table: GSEA Results Summary

  

Fig 1: Enrichment plot: HALLMARK\_INTERFERON\_GAMMA\_RESPONSE      
 Profile of the Running ES Score & Positions of GeneSet Members on the Rank Ordered List

  

| PROBE | DESCRIPTION (from dataset) | GENE SYMBOL | GENE\_TITLE | RANK IN GENE LIST | RANK METRIC SCORE | RUNNING ES | CORE ENRICHMENT || 1 | LYSMD2 | na |  |  | 155 | 2.198 | 0.0059 | No |
| 2 | MTHFD2 | na |  |  | 172 | 2.163 | 0.0189 | No |
| 3 | PNPT1 | na |  |  | 320 | 1.926 | 0.0235 | No |
| 4 | TRIM14 | na |  |  | 363 | 1.877 | 0.0333 | No |
| 5 | SLAMF7 | na |  |  | 493 | 1.774 | 0.0379 | No |
| 6 | PNP | na |  |  | 546 | 1.742 | 0.0463 | No |
| 7 | CD274 | na |  |  | 836 | 1.574 | 0.0412 | No |
| 8 | BANK1 | na |  |  | 987 | 1.517 | 0.0430 | No |
| 9 | CASP3 | na |  |  | 1155 | 1.458 | 0.0436 | No |
| 10 | IFIT1 | na |  |  | 1463 | 1.358 | 0.0361 | No |
| 11 | PTPN1 | na |  |  | 1865 | 1.253 | 0.0231 | No |
| 12 | ISG15 | na |  |  | 1964 | 1.231 | 0.0258 | No |
| 13 | USP18 | na |  |  | 2289 | 1.159 | 0.0162 | No |
| 14 | LATS2 | na |  |  | 2335 | 1.148 | 0.0212 | No |
| 15 | NUP93 | na |  |  | 2356 | 1.143 | 0.0274 | No |
| 16 | PARP12 | na |  |  | 2539 | 1.104 | 0.0249 | No |
| 17 | IL18BP | na |  |  | 2574 | 1.098 | 0.0302 | No |
| 18 | UPP1 | na |  |  | 2597 | 1.095 | 0.0360 | No |
| 19 | TRIM25 | na |  |  | 2663 | 1.082 | 0.0395 | No |
| 20 | RSAD2 | na |  |  | 3298 | 0.971 | 0.0124 | No |
| 21 | SAMHD1 | na |  |  | 3369 | 0.959 | 0.0149 | No |
| 22 | PTPN2 | na |  |  | 3585 | 0.923 | 0.0095 | No |
| 23 | CASP8 | na |  |  | 3766 | 0.890 | 0.0057 | No |
| 24 | HERC6 | na |  |  | 4081 | 0.839 | -0.0054 | No |
| 25 | ISOC1 | na |  |  | 4105 | 0.836 | -0.0013 | No |
| 26 | ARL4A | na |  |  | 4169 | 0.826 | 0.0007 | No |
| 27 | SRI | na |  |  | 4299 | 0.808 | -0.0009 | No |
| 28 | PSMA3 | na |  |  | 4449 | 0.785 | -0.0037 | No |
| 29 | LAP3 | na |  |  | 4650 | 0.756 | -0.0094 | No |
| 30 | IFI44L | na |  |  | 4958 | 0.713 | -0.0209 | No |
| 31 | PELI1 | na |  |  | 5111 | 0.691 | -0.0245 | No |
| 32 | SAMD9L | na |  |  | 5166 | 0.682 | -0.0230 | No |
| 33 | MARCH1 | na |  |  | 5241 | 0.673 | -0.0226 | No |
| 34 | STAT1 | na |  |  | 5448 | 0.645 | -0.0293 | No |
| 35 | CASP7 | na |  |  | 5606 | 0.625 | -0.0335 | No |
| 36 | PSMA2 | na |  |  | 5669 | 0.614 | -0.0328 | No |
| 37 | EIF2AK2 | na |  |  | 6165 | 0.547 | -0.0553 | No |
| 38 | RTP4 | na |  |  | 6227 | 0.536 | -0.0551 | No |
| 39 | FAS | na |  |  | 6266 | 0.532 | -0.0537 | No |
| 40 | JAK2 | na |  |  | 6438 | 0.510 | -0.0594 | No |
| 41 | NOD1 | na |  |  | 6548 | 0.496 | -0.0620 | No |
| 42 | SPPL2A | na |  |  | 6605 | 0.492 | -0.0618 | No |
| 43 | NMI | na |  |  | 6667 | 0.482 | -0.0619 | No |
| 44 | SP110 | na |  |  | 6804 | 0.467 | -0.0661 | No |
| 45 | GCH1 | na |  |  | 6881 | 0.459 | -0.0671 | No |
| 46 | TDRD7 | na |  |  | 6947 | 0.451 | -0.0677 | No |
| 47 | IFIT3 | na |  |  | 7001 | 0.447 | -0.0676 | No |
| 48 | EIF4E3 | na |  |  | 7242 | 0.416 | -0.0775 | No |
| 49 | PSME2 | na |  |  | 7980 | 0.328 | -0.1142 | No |
| 50 | PSMB2 | na |  |  | 8011 | 0.324 | -0.1137 | No |
| 51 | IRF2 | na |  |  | 8035 | 0.322 | -0.1128 | No |
| 52 | SSPN | na |  |  | 8214 | 0.303 | -0.1202 | No |
| 53 | PLSCR1 | na |  |  | 8332 | 0.290 | -0.1245 | No |
| 54 | IFIT2 | na |  |  | 8545 | 0.266 | -0.1340 | No |
| 55 | TRIM26 | na |  |  | 8599 | 0.261 | -0.1351 | No |
| 56 | RAPGEF6 | na |  |  | 8673 | 0.250 | -0.1373 | No |
| 57 | IFI44 | na |  |  | 8902 | 0.223 | -0.1479 | No |
| 58 | SLC25A28 | na |  |  | 9831 | 0.123 | -0.1958 | No |
| 59 | FGL2 | na |  |  | 10347 | 0.062 | -0.2225 | No |
| 60 | CASP1 | na |  |  | 10391 | 0.060 | -0.2244 | No |
| 61 | NLRC5 | na |  |  | 10739 | 0.020 | -0.2425 | No |
| 62 | ZNFX1 | na |  |  | 10743 | 0.020 | -0.2425 | No |
| 63 | CMPK2 | na |  |  | 10862 | 0.006 | -0.2487 | No |
| 64 | CASP4 | na |  |  | 10964 | 0.000 | -0.2540 | No |
| 65 | ADAR | na |  |  | 11005 | -0.004 | -0.2561 | No |
| 66 | NFKB1 | na |  |  | 11078 | -0.014 | -0.2597 | No |
| 67 | HIF1A | na |  |  | 11136 | -0.018 | -0.2626 | No |
| 68 | CFH | na |  |  | 11540 | -0.062 | -0.2834 | No |
| 69 | TOR1B | na |  |  | 11640 | -0.076 | -0.2881 | No |
| 70 | NCOA3 | na |  |  | 11832 | -0.104 | -0.2975 | No |
| 71 | CD40 | na |  |  | 11978 | -0.123 | -0.3043 | No |
| 72 | DDX60 | na |  |  | 11988 | -0.124 | -0.3040 | No |
| 73 | WARS | na |  |  | 12021 | -0.127 | -0.3049 | No |
| 74 | PLA2G4A | na |  |  | 12476 | -0.181 | -0.3276 | No |
| 75 | BPGM | na |  |  | 12551 | -0.191 | -0.3302 | No |
| 76 | STAT3 | na |  |  | 12572 | -0.194 | -0.3300 | No |
| 77 | OAS3 | na |  |  | 12687 | -0.211 | -0.3347 | No |
| 78 | RBCK1 | na |  |  | 12706 | -0.214 | -0.3343 | No |
| 79 | RIPK1 | na |  |  | 12747 | -0.221 | -0.3349 | No |
| 80 | TRIM21 | na |  |  | 13344 | -0.295 | -0.3644 | No |
| 81 | IL7 | na |  |  | 13349 | -0.295 | -0.3627 | No |
| 82 | HLA-DQA1 | na |  |  | 13363 | -0.297 | -0.3615 | No |
| 83 | IFNAR2 | na |  |  | 13468 | -0.310 | -0.3650 | No |
| 84 | MX1 | na |  |  | 13486 | -0.314 | -0.3638 | No |
| 85 | MYD88 | na |  |  | 13595 | -0.329 | -0.3674 | No |
| 86 | RNF31 | na |  |  | 13607 | -0.331 | -0.3659 | No |
| 87 | IFI30 | na |  |  | 13832 | -0.356 | -0.3754 | No |
| 88 | CXCL10 | na |  |  | 13845 | -0.359 | -0.3737 | No |
| 89 | IL15RA | na |  |  | 14050 | -0.385 | -0.3820 | No |
| 90 | OASL | na |  |  | 14184 | -0.403 | -0.3864 | No |
| 91 | MT2A | na |  |  | 14804 | -0.498 | -0.4157 | No |
| 92 | IRF7 | na |  |  | 14815 | -0.500 | -0.4130 | No |
| 93 | VCAM1 | na |  |  | 15075 | -0.525 | -0.4233 | No |
| 94 | PTPN6 | na |  |  | 15123 | -0.532 | -0.4223 | No |
| 95 | ITGB7 | na |  |  | 15259 | -0.556 | -0.4259 | No |
| 96 | CCL2 | na |  |  | 15276 | -0.560 | -0.4231 | No |
| 97 | RIPK2 | na |  |  | 15343 | -0.573 | -0.4229 | No |
| 98 | RNF213 | na |  |  | 15348 | -0.573 | -0.4195 | No |
| 99 | PFKP | na |  |  | 15615 | -0.609 | -0.4296 | No |
| 100 | IFIH1 | na |  |  | 15792 | -0.647 | -0.4347 | Yes |
| 101 | XAF1 | na |  |  | 15802 | -0.649 | -0.4310 | Yes |
| 102 | IL15 | na |  |  | 15840 | -0.654 | -0.4288 | Yes |
| 103 | PML | na |  |  | 15940 | -0.675 | -0.4296 | Yes |
| 104 | CDKN1A | na |  |  | 15950 | -0.678 | -0.4258 | Yes |
| 105 | TRAFD1 | na |  |  | 15985 | -0.683 | -0.4232 | Yes |
| 106 | SOCS3 | na |  |  | 16002 | -0.686 | -0.4196 | Yes |
| 107 | ST8SIA4 | na |  |  | 16024 | -0.691 | -0.4163 | Yes |
| 108 | DDX58 | na |  |  | 16198 | -0.731 | -0.4207 | Yes |
| 109 | OAS2 | na |  |  | 16219 | -0.737 | -0.4171 | Yes |
| 110 | IRF9 | na |  |  | 16304 | -0.759 | -0.4166 | Yes |
| 111 | IFI35 | na |  |  | 16370 | -0.774 | -0.4151 | Yes |
| 112 | SOCS1 | na |  |  | 16397 | -0.781 | -0.4115 | Yes |
| 113 | UBE2L6 | na |  |  | 16481 | -0.806 | -0.4107 | Yes |
| 114 | OGFR | na |  |  | 16562 | -0.826 | -0.4096 | Yes |
| 115 | VAMP5 | na |  |  | 16588 | -0.832 | -0.4056 | Yes |
| 116 | LY6E | na |  |  | 16861 | -0.908 | -0.4141 | Yes |
| 117 | PSME1 | na |  |  | 16865 | -0.910 | -0.4084 | Yes |
| 118 | PIM1 | na |  |  | 17218 | -1.013 | -0.4204 | Yes |
| 119 | PSMB8 | na |  |  | 17300 | -1.042 | -0.4180 | Yes |
| 120 | ARID5B | na |  |  | 17386 | -1.071 | -0.4156 | Yes |
| 121 | IFITM3 | na |  |  | 17388 | -1.072 | -0.4088 | Yes |
| 122 | BATF2 | na |  |  | 17393 | -1.073 | -0.4022 | Yes |
| 123 | TAP1 | na |  |  | 17413 | -1.084 | -0.3962 | Yes |
| 124 | SECTM1 | na |  |  | 17427 | -1.093 | -0.3899 | Yes |
| 125 | NFKBIA | na |  |  | 17437 | -1.096 | -0.3834 | Yes |
| 126 | IFITM2 | na |  |  | 17476 | -1.113 | -0.3782 | Yes |
| 127 | C1R | na |  |  | 17490 | -1.119 | -0.3718 | Yes |
| 128 | TNFAIP3 | na |  |  | 17494 | -1.121 | -0.3647 | Yes |
| 129 | PSMB10 | na |  |  | 17606 | -1.151 | -0.3632 | Yes |
| 130 | CFB | na |  |  | 17623 | -1.158 | -0.3566 | Yes |
| 131 | TAPBP | na |  |  | 17642 | -1.164 | -0.3501 | Yes |
| 132 | LGALS3BP | na |  |  | 17867 | -1.260 | -0.3539 | Yes |
| 133 | MVP | na |  |  | 17873 | -1.261 | -0.3460 | Yes |
| 134 | ICAM1 | na |  |  | 17954 | -1.306 | -0.3419 | Yes |
| 135 | IRF1 | na |  |  | 18023 | -1.342 | -0.3369 | Yes |
| 136 | HLA-G | na |  |  | 18076 | -1.368 | -0.3309 | Yes |
| 137 | IL4R | na |  |  | 18107 | -1.380 | -0.3236 | Yes |
| 138 | STAT2 | na |  |  | 18119 | -1.384 | -0.3153 | Yes |
| 139 | PSMB9 | na |  |  | 18166 | -1.407 | -0.3088 | Yes |
| 140 | FPR1 | na |  |  | 18271 | -1.474 | -0.3048 | Yes |
| 141 | PDE4B | na |  |  | 18287 | -1.482 | -0.2961 | Yes |
| 142 | PARP14 | na |  |  | 18354 | -1.529 | -0.2898 | Yes |
| 143 | CD74 | na |  |  | 18418 | -1.591 | -0.2829 | Yes |
| 144 | NAMPT | na |  |  | 18422 | -1.594 | -0.2729 | Yes |
| 145 | TNFSF10 | na |  |  | 18488 | -1.633 | -0.2658 | Yes |
| 146 | ISG20 | na |  |  | 18505 | -1.647 | -0.2561 | Yes |
| 147 | HLA-B | na |  |  | 18532 | -1.675 | -0.2468 | Yes |
| 148 | ST3GAL5 | na |  |  | 18542 | -1.683 | -0.2365 | Yes |
| 149 | TXNIP | na |  |  | 18572 | -1.710 | -0.2271 | Yes |
| 150 | APOL6 | na |  |  | 18574 | -1.715 | -0.2162 | Yes |
| 151 | B2M | na |  |  | 18622 | -1.775 | -0.2073 | Yes |
| 152 | PTGS2 | na |  |  | 18633 | -1.789 | -0.1963 | Yes |
| 153 | CIITA | na |  |  | 18741 | -1.952 | -0.1895 | Yes |
| 154 | MX2 | na |  |  | 18748 | -1.963 | -0.1772 | Yes |
| 155 | HLA-DMA | na |  |  | 18758 | -1.979 | -0.1650 | Yes |
| 156 | IL6 | na |  |  | 18776 | -2.020 | -0.1530 | Yes |
| 157 | HLA-DRB1 | na |  |  | 18792 | -2.049 | -0.1407 | Yes |
| 158 | BTG1 | na |  |  | 18836 | -2.149 | -0.1292 | Yes |
| 159 | BST2 | na |  |  | 18856 | -2.210 | -0.1161 | Yes |
| 160 | CMKLR1 | na |  |  | 18878 | -2.270 | -0.1026 | Yes |
| 161 | C1S | na |  |  | 18893 | -2.301 | -0.0887 | Yes |
| 162 | DHX58 | na |  |  | 18900 | -2.322 | -0.0741 | Yes |
| 163 | GBP4 | na |  |  | 18924 | -2.365 | -0.0602 | Yes |
| 164 | SOD2 | na |  |  | 18970 | -2.521 | -0.0464 | Yes |
| 165 | VAMP8 | na |  |  | 19018 | -2.739 | -0.0314 | Yes |
| 166 | IFI27 | na |  |  | 19088 | -3.055 | -0.0155 | Yes |
| 167 | CXCL11 | na |  |  | 19118 | -3.311 | 0.0042 | Yes |
Table: GSEA details [plain text format]

  

Fig 2: HALLMARK\_INTERFERON\_GAMMA\_RESPONSE      
 Blue-Pink O' Gram in the Space of the Analyzed GeneSet

  

Fig 3: HALLMARK\_INTERFERON\_GAMMA\_RESPONSE: Random ES distribution      
 Gene set null distribution of ES for **HALLMARK\_INTERFERON\_GAMMA\_RESPONSE**
